# Bilateral Meningioma: A Case Report and Review of the Literature

**DOI:** 10.3390/ijms23031187

**Published:** 2022-01-21

**Authors:** Anja Bukovac, Hana Panić, Tomislava Mrgan, Nika Šlaus, Anja Kafka, Niko Njirić, Nives Pećina-Šlaus

**Affiliations:** 1 Laboratory of Neuro-Oncology, Croatian Institute for Brain Research, School of Medicine, University of Zagreb, Šalata 12, 10000 Zagreb, Croatia; anja.kafka@mef.hr (A.K.); nives.pecina.slaus@mef.hr (N.P.-Š.); 2 Department of Biology, School of Medicine, University of Zagreb, Šalata 3, 10000 Zagreb, Croatia; 3 School of Medicine, University of Zagreb, Šalata 3, 10000 Zagreb, Croatia; hanaapanic@gmail.com (H.P.); tomislava.mrgan@gmail.com (T.M.); slausnik@gmail.com (N.Š.); 4 Department of Neurosurgery, University Hospital Center “Zagreb”, School of Medicine, University of Zagreb, Kišpatićeva 12, 10000 Zagreb, Croatia; njiricn@gmail.com

**Keywords:** multiple meningioma, bilateral meningioma, EMT, Wnt signaling, E-cadherin, N-cadherin, SNAIL&SLUG, TWIST1, beta-catenin, GSK3beta, DVL1

## Abstract

Here, we present a rarely seen example of bilateral meningiomas exhibiting different malignancy grades, I (meningothelial) and II (atypical), recorded in a 72-year-old patient. The presence of two separated lesions of different grades in a single patient can elucidate meningioma progression. To this end, the involvement of specific protein markers of epithelial to mesenchymal transition (EMT), the process responsible for progression, was tested in both tumors. Protein expression status of specific epithelial (E-cadherin) and mesenchymal markers (N-cadherin, SNAIL&SLUG and TWIST1) was investigated. Furthermore, markers that are connected to Wnt signaling pathway–beta-catenin, GSK3beta and DVL1—were also analyzed. For signs of neurofibromatosis and schwanomatosis genetic testing was performed. Immunohistochemistry evaluated by immunoreactivity score (IRS) was used to determine the signal strengths and proteins’ location. Our results indicated that, in comparison to the grade I tumor, mesenchymal markers SNAIL and SLUG were upregulated in the atypical meningioma. TWIST1, beta-catenin and GSK3beta were upregulated in both grades, while E-cadherin was partially lost. A pronounced cadherin switch could not be established; however, N-cadherin showed widespread tissue presence. Genetic testing did not detect changes of *NF2* or *SMARCB1* genes denying germline origin of the lesions. The rare presence of two different grades in one patient elucidate previously unknown molecules involved in meningioma progression.

## 1. Introduction

Meningiomas are the most common primary intracranial benign tumors developed from arachnoid cells [1,2,3]. Tumors are commonly diagnosed in the elderly (median age 65 years) with a female to male ratio of 3:1. The main predisposing factors for meningioma development are inactivation of the *NF2* gene and exposure to ionizing radiation, which can be causative for multiple neoplasms [1,2]. The majority of meningiomas are slow-growing benign (grade I), while atypical (grade II) are less common, representing approximately 10–15%. Anaplastic (grade III) show malignant phenotype, representing 3% of meningiomas [2,4,5]. Meningiomas can be additionally divided into 15 subtypes based on their histology, where grade I contains nine subtypes, while grades II and III each contain three [2]. Regarding the greater tendency to relapse and metastasize, grade II and III meningiomas have five-year survival rate below 60%, compared to grade I of 80% [1,2]. Rates of relapse for atypical are 29–52% and for anaplastic 50–94% [6]. Therapy for meningiomas is complete excision [1]. Radiotherapy may be used in some cases of atypical and anaplastic meningiomas when the tumor is not completely resectable or is located on the cranial base [1,3,7]. Although meningiomas typically present as sporadic solitary lesions, up to 10% of patients present multiple meningiomas [8,9,10,11,12,13]. Multiple meningiomas are diagnosed when two or more spatially separated lesions are found. They can arise as a consequence of *NF2* germline mutational inherent pattern, or as sporadic non-hereditary cases [14]. However, the etiology of multiple meningioma is still controversial [15]. There are two views regarding the arise of multiple meningiomas, one is that they occur independently and the other is that they arise as the result of subsequent propagation of single clonal expansion.

The case investigated here is a patient with bilateral meningiomas of different grades. Such simultaneous occurrences happen rarely, only in one-third of multiple meningiomas [16] making this case interesting. Because of different malignancy grades, we decided to investigate the involvement of specific epithelial to mesenchymal transition (EMT) protein markers in both tumors. EMT is responsible for tumor progression and malignancy and is an important indicator of cellular mobility acquisition. Special emphasis was given to markers connected to Wnt signaling, since it has been largely documented that the Wnt pathway plays an important role in EMT programs [4].

We specifically searched for changes of E-cadherin and N-cadherin, molecules that represent the so-called cadherin-switch in EMT, as well as SNAIL&SLUG and TWIST1, which are well-known EMT transcription factors and beta-catenin, a marker of mesenchymal phenotype. Additionally, we studied GSK3beta and DVL1, whose roles are still unknown in EMT, and tried to define them in bilateral meningioma.

## 2. Case Description

**Figure 1:**
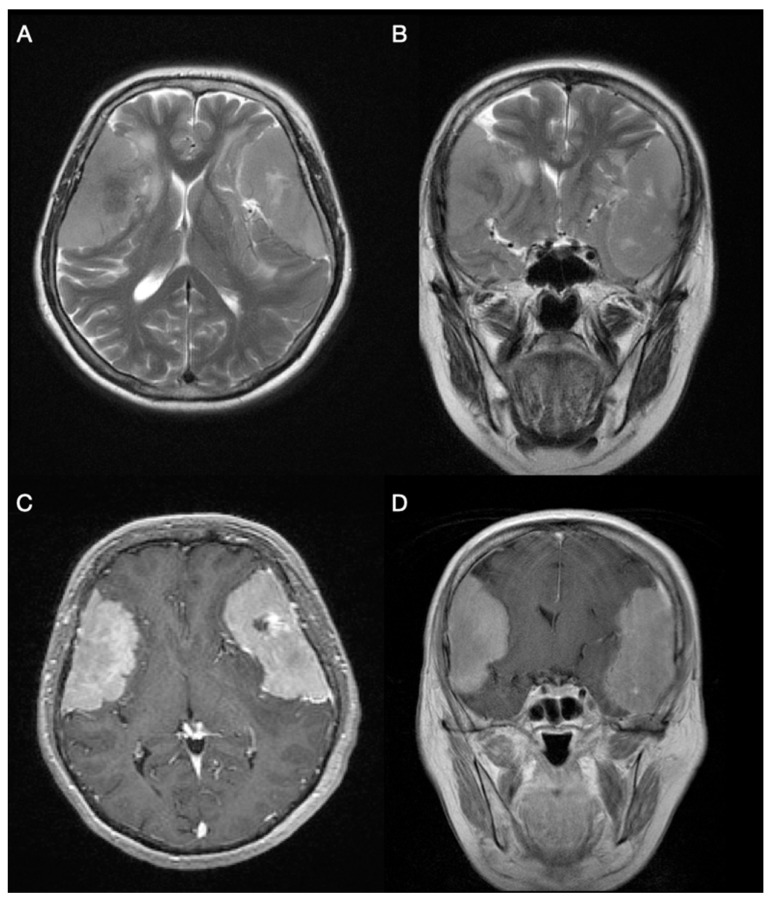
Bilateral, large frontotemporoparietal convexity meningiomas are shown on axial (**A**) and coronal (**B**) T2-weighted magnetic resonance imaging, with minimal signs of peritumoral edema. T1-weighted gadolinium-enhanced MRI axial (**C**) and coronal (**D**) images display homogenous contrast enhancement and bilateral “dural tail” signs.

A 72-year-old female from Croatia, suffering from the bilateral meningioma, was admitted to hospital due to bradykinesia, loss of memory and communication difficulties. The patient also showed impaired mobility and required assistive devices. At admission, the patient was alert, attentive and less oriented to time, place and person, GCS = 14, MMSE = 7. Her pupils were equal, round and reactive to light and accommodation. There were no abnormalities in eye movement. Functions of other cranial nerves were intact. Muscle strength of upper and lower extremities was preserved. MRI following an MSCT scan of the brain showed lobulated expansive masses bilaterally in the frontotemporoparietal region with local edema and compression of surrounding cerebral parenchyma (Figure 1). Surgical treatment was appointed in Hospital Center Zagreb; the first one in December 2017 and the second one in February 2018.

In 2017, osteoplastic pterional craniotomy and meningioma ablation were performed in the left frontotemporoparietal region. Surgery went as planned and the patient’s postoperative state was well. The results of the PHD analysis are shown in Table 1.

A postoperative brain MRI was performed showing subdural liquid collection of great density with a maximum diameter of 13 mm and marginal imbibition. In the adjacent frontal lobe cortex and temporal operculum, there were signs of gliosis and cortical laminar necrosis. In the right frontotemporoparietal region and near to the greater wing of the sphenoid bone, a meningioma was seen with mild perifocal edema and significant compression of the frontal and temporal lobe parenchyma as well as of the ventricular system. In addition, mild subfalcine and uncal herniation was present. There were no signs of acute ischemia or bleeding. Left mastoid cells were filled with liquid.

During the clinical exam before the second surgery, the patient reported some cognitive issues, with no other symptoms that were reported during first hospitalization in 2017. At admission, the patient was alert, attentive and less oriented to time, place and person, GCS = 14, MMSE = 7, with significant cognitive impairment and semantic dysphasia. Extraocular eye movement was intact with no nystagmus present. The pupils were equal, round and reactive to light and accommodation. Functions of other cranial nerves were intact. Muscle strength of upper and lower extremities was preserved. Heel and toe walking could not be performed. There was a normal and symmetric response of patellar reflex and reflex of the Achilles tendon, with no pathological reflexes. Sensory function was preserved. In the anti-gravitational test patient managed to maintain the initial position of upper extremities, the Mingazzini test could not be executed. There was no dysmetria in coordination tests. Meningeal signs were negative. Sphincter control was intact.

In January 2018, osteoplastic pterional craniotomy and meningioma ablation were performed in the right frontotemporoparietal region. The additional tumor was removed completely using microsurgical techniques and sent to PHD analysis (shown in Table 2).

After surgery, an external lumbar drain was placed and the patient was put in an ICU. A brain MSCT showed a 5 cm hematoma located in the frontal lobe and a few smaller ones located in the right temporal lobe. Perifocal edema and hematomas caused significant subfalcine herniation with 15 mm midline shift to the left. Emergency hematoma evacuation was indicated. After the operation, the patient’s neurological status was improved and the electrolyte imbalance, hypoglycemia and hypoalbuminemia were also corrected. A brain CT scan showed mild progression of edema and bleeding in the right frontal region with greater expansive effect on the right lateral ventricle. The patient was transferred to the post-intensive care unit. A brain CT scan showed partial decomposition of the right temporal hematoma and significant regression of the brain edema. There were no signs of acute ischemia, bleeding or hydrocephalus.

The tumor samples together with corresponding autologous blood were collected with the patients’ consent from the Department of Neurosurgery and Department of Pathology, University Hospital Center “Zagreb”. Both tumors were studied by certified neuropathologist and classified according to the WHO criteria [17]. The patient had no known family history of brain tumors and did not undergo any cancer treatment prior to surgery.

## 3. Results

### 3.1. Sequence Analysis and Deletion/Duplication Testing of NF2 and SMARCB1 Genes

The reason for genetic analyses was to establish the germline mutation in a patient regarding neurofibromatosis in which bilateral meningioma are common or regarding potential schwannomatosis. The genetic testing did not reveal common pathogenic variants of *NF2* and *SMARCB1* genes known to cause the disease. No reportable genetic variants of known significance were identified by sequence and deletion/duplication analyses, indicating that this was not a case of familial neurofibromatosis or schwanomatosis. However, this finding should be taken with caution, since promoters, untranslated regions, and other non-coding regions were not interrogated and only targeted loci were analyzed. Other genetic analyses and polymorphisms were not included in our study. However, we have performed additional analyses and found one benign variant in the *SMARCB1* gene NM_003073.3:c.897G > A (silent, heterozygous, frequency in population: 11.40%), which we included in the manuscript.

### 3.2. Levels of E- and N-Cadherins Expression

**Figure 2:**
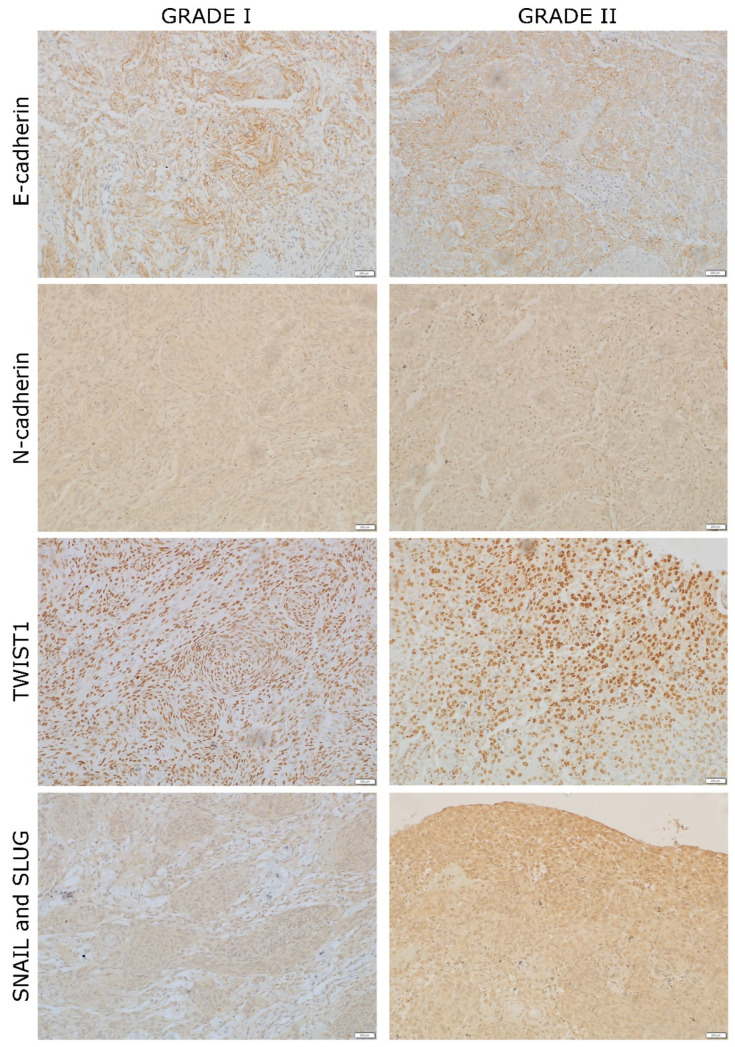
Example of bilateral meningioma grade I and II hotspots stained for EMT mediators: E-cadherin, N-cadherin, TWIST1, SNAIL and SLUG. The hotspots are shown at 200× magnification.

The difference between major marker of the epithelial phenotype—E-cadherin—and the major marker of the mesenchymal phenotype—N-cadherin—was established in our patient’s tumors. Grade I tumor was mostly negative to E-cadherin with approximately 5% of tissue showing expression with hotspot IRS value 9, while grade II tumor showed broader tissue areas of E-cadherin expression in about 50% of examined tissue with hotspot IRS value 12. On the other hand, levels of N-cadherin were low (1+) in both tumors and the protein was expressed equally through the whole tissue (IRS = 4) (Figure 2).

### 3.3. Levels of TWIST1, SNAIL and SLUG Expression

The high expression levels of all three investigated transcription factors involved in EMT were noted. Both grade I and II showed several hotspots with high expression (3+) (IRS = 12) of TWIST1, while substantial area of tumor tissue showed low (1+) TWIST1 nuclear expression. SNAIL and SLUG were expressed in cytoplasm in 20% of both grade I and II tumor. Grade II showed higher intensity with IRS value 12, while grade I hotspots had IRS value 8. Additionally, the nuclear expression was more frequent in grade II (Figure 2).

### 3.4. Levels of Beta-Catenin and GSK3beta Expression

**Figure 3:**
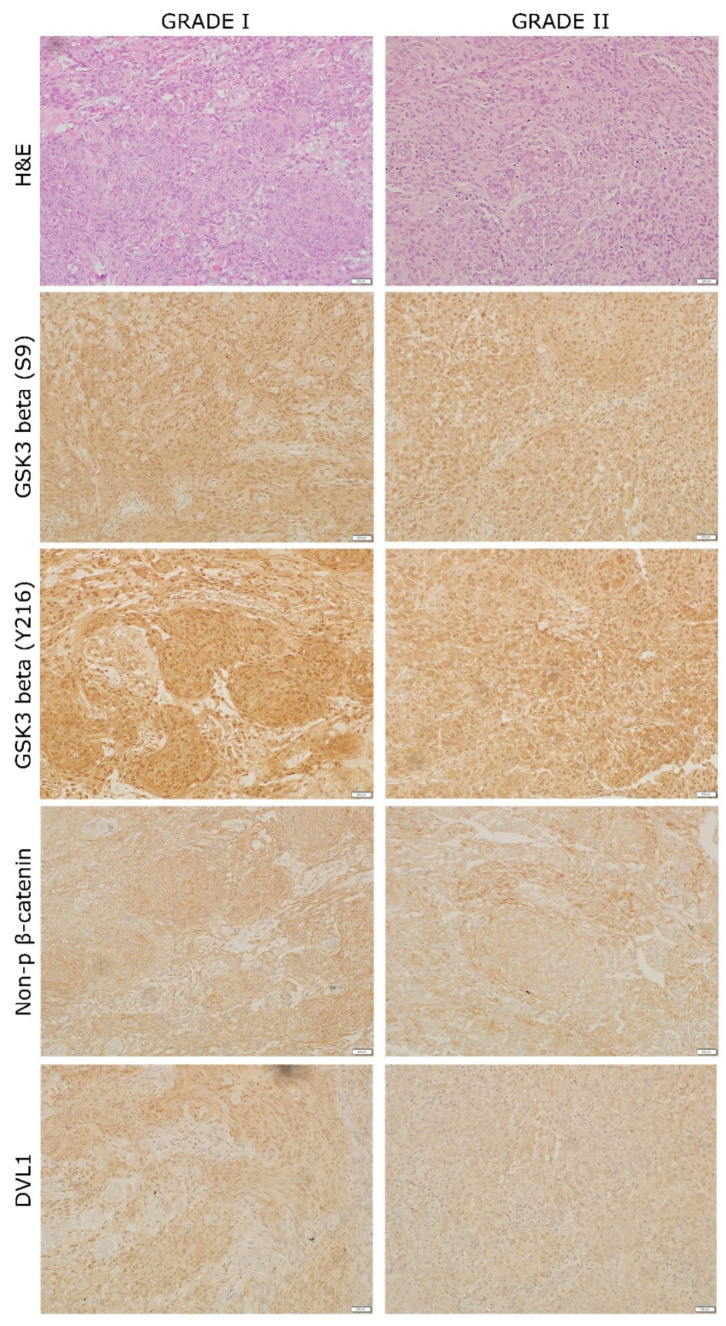
Example of bilateral meningioma grade I and II hotspots stained for hematoxylin and eosin and Wnt signaling mediators: GSK3beta (S9), GSK3beta (Y216), non-phospho (active) beta-catenin and DVL1. The hotspots are shown at 200× magnification.

Wnt signaling is involved in EMT and tumor progression scenarios and the results for the two main signaling molecules, beta-catenin and GSK3beta, showed distinct patterns. Beta-catenin was investigated with two antibodies, one of which detects total beta-catenin and the other detects just its active form. Total beta-catenin was highly expressed in 95% of both tumors (IRS = 12) and located mostly in the membrane. However, when analyzing the active form, the expression dropped in the majority of cells in both tumors but with different hotspot intensity. Hotspots in grade I showed more pronounced expression (IRS = 12), while hotspots in grade II showed an IRS value of 8. No nuclei expression was noted (Figure 3).

Two forms of GSK3beta were investigated using antibodies that detect its active (GSK3beta Y216) and inactive (GSK3beta S9) form. Both forms were highly expressed in both tumors with hotspot IRS value 12. Protein expression was detected in cytoplasm and nuclei. A high expression of the active form of GSK3beta was found in 50% of grade I and grade II tumor tissue, while the inactive form of GSK3beta was overall more pronounced than the active form and showed high expression in 60% of grade I tissue and 80% of grade II tissue (Figure 3).

### 3.5. Levels of DVL1 Expression

The central mediator of Wnt signaling DVL1, which inhibits the beta-catenin degradation was detected in the nuclei and cytoplasm. Grade I revealed expression in only 20% of its tissue, but with a higher IRS hotspot value (IRS = 8). Additionally, 90% of nuclei in the hotspot showed high DVL1 expression. On the other hand, grade II had a protein expression in about 80% of the tissue but with lower IRS hotspot value (IRS = 4). In grade II, the hotspot nuclear expression was also lower, with 76% of nuclei showing immunopositivity (Figure 3).

**Figure 4:**
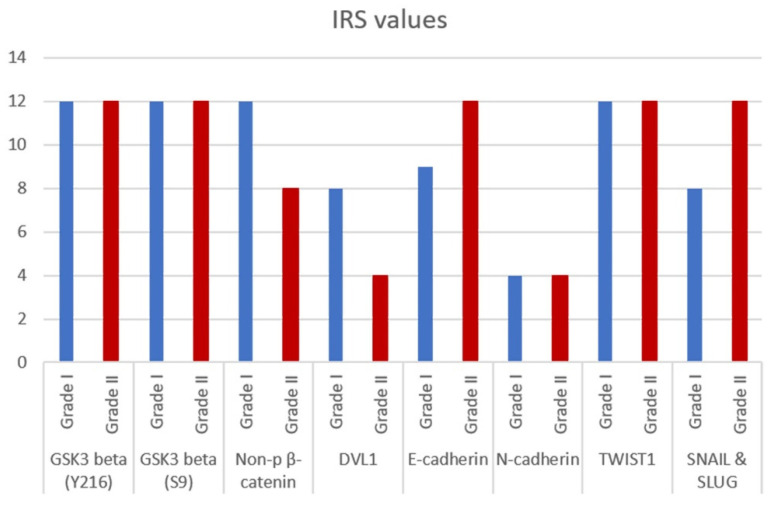
IRS values of different grades (I and II) of bilateral meningioma hotspots for GSK3beta (Y216), GSK3beta (S9), non-phospho (active) beta-catenin, DVL1, E-cadherin, N-cadherin, TWIST1 and SNAIL and SLUG.

The expression levels for all investigated proteins are shown in Figure 4.

## 4. Discussion

The rare incidence of bilateral meningiomas, and especially those with different histology, are useful for studying progression. The most common histological types found in multiple meningiomas include fibroblastic, meningothelial, psammomatous and transitional types [17]. Grade I tumor that we report here was of the meningothelial type, while grade II was atypical. It has been suggested that multiple meningiomas do not differ in prognosis, clinical features or histology from the solitary types, and are not considered a specific entity [18,19]. However, knowledge on multiple meningiomas remains inadequately explained [6]. New findings of molecular characteristics of meningiomas have offered more accurate grading and prediction of prognosis and recurrence [20,21,22]. Contrary, for multiple meningiomas, the detection of molecular changes and potential biomarkers are still missing.

Reports on non-*NF2* mutated meningioma showed relatively low frequency of genomic alterations per patient. One of the most common mutations are those of the *TRAF7* gene. They are mutually exclusive to *NF2* mutations but can usually be found in combination with mutations in *AKT1* or in *KLF4*. Additionally, mutations that occur in the *SMARCB1* gene, which were investigated in our patient, are reported as part of the genetic profile of non-*NF2* meningioma [23]. One must also consider polymorphisms as contributors to meningioma genetic profile; for instance, we have encountered a benign variant NM_003073.3:c.897G > A of the *SMARCB1* gene with a population frequency of 11.40%. Although we have not found disease-associated variants in *NF2* and *SMARCB1* genes, it is still possible that undefined variants may contribute to meningioma recurrence. For example, other polymorphisms have been reported to have a role in meningioma development, such as those in *NFKB1, VCAM1, FCER1G, CD14, TNFRSF18, RAC2, XDH, C1D, CCR6, TLR1/TLR10/TLR6*, *NOS1,* and *DEFA5* genes [24].

Ideally, we would have liked to deepen the investigation on genetic inheritance mechanisms that are different from the two genes that we studied, employing targeted NGS or WES, but because of financial constraints, we were not afforded this opportunity. We performed a basic rudimentary clinical diagnostic test that was unable to determine a hereditary component to the disease. Promoter changes, non-coding exons and intronic variants were not covered by this test. Naturally, a more detailed genetic analysis on other candidate genes and mutation types may have provided different results. It is important to understand how meningioma progresses. Our group has been involved in EMT and WNT signaling and the case of bilateral meningioma that we found was ideal for the investigation of meningioma progression.

Here, we show the lack of E-cadherin in at least 50% of tumor tissue. In spite of the fact that we could not establish a pronounced loss of this protein in grade II tumor, it was still obvious that substantial parts of both tumors lost E-cadherin. The cadherin switch was not very pronounced, either. However, the expression of mesenchymal phenotype marker N-cadherin was present and equally distributed in most cells of both investigated meningiomas. These findings could be interpreted with partial EMT, but also with the fact that cadherin switch in tumor progression need not involve these particular cadherins. There are other possible candidates that were reported to be changed in the process of EMT, for instance P-cadherin, but this needs to be further investigated. There are indications that successful tumor invasion is the result of partial EMT where the expression of epithelial and mesenchymal markers is simultaneous [25,26]. In such a spatial fashion, tumors acquire plasticity that allows them to adapt to the new microenvironment.

Transcription factors involved in EMT clearly showed elevated levels of expression in cytoplasm as well as nuclei. TWIST1 was slightly more prevalent in grade II, while SNAIL and SLUG were much stronger and frequent in grade II tumors, indicating their association to meningioma progression. This is consistent with other studies that report on SNAIL and SLUG upregulation in higher grades. Such transcription factors suppress the expression of E-cadherin by binding to its promoter and stopping its transcription [27].

Furthermore, the high levels of beta-catenin were also indicative of EMT. We have shown that cytoplasmic stability of this protein is constant with the high presence of its active form. Beta-catenin is an important marker of mesenchymal phenotype. It has been reported that the upregulation of beta-catenin can activate the transcriptional repressors SNAIL and SLUG and, through the downregulation of E-cadherin, induce EMT, which is compatible with the results of this investigation [25].

High levels of active beta-catenin can be maintained by DVL1, the expression of which was more widespread in grade II. Additionally, detected nuclear activity of DVL1 suggests its role in activation of transcription of Wnt target genes [28].

Both forms of GSK3beta were highly expressed in our tumors. The expression of the inactive form increased in grade II tumor, which may lead to impaired destruction of beta-catenin and cell proliferation. GSK3beta has diverse roles in numerous cellular processes and can display both pro-oncogenic and tumor-suppressive effects.

Our results regarding selected proteins are novel for bilateral meningioma. However, the selected molecules were investigated in solitary cases by many authors, including our group. Previously [29,30], we have found the increase in N-cadherin expression in relation to E-cadherin. Additionally, transcription factors SNAIL, SLUG and TWIST1 were stronger than E- and N-cadherin, and SNAIL and SLUG were significantly associated with higher grades. The present study shows that although a pronounced cadherin switch could not be detected, the basis for EMT and activation of Wnt signaling is established.

There are several novel articles investigating meningioma invasion [5,31,32] that described novel molecules and epigenetic events, and our present findings can contribute further with new candidates.

In conclusion, the results of this study contribute to bilateral meningioma genetic blueprint and indicate that key players of EMT and Wnt pathway have specific roles in bilateral meningioma.

## 5. Methods

### 5.1. DNA Extraction and Genetic Testing

DNA extraction from blood tissue was performed using standard protocol described previously [29]. Briefly, the salting-out method by isopropanol precipitation was used to obtain DNA from leukocytes. Genetic testing for *NF2* and *SMARCB1*, which included sequence analysis and deletion/duplication testing, was performed by Invitae Corporation (San Francisco, CA, USA). Genomic DNA obtained from the submitted sample was enriched for targeted regions using a hybridization-based protocol and sequenced using Illumina technology. All targeted regions were sequenced with ≥50× depth or were supplemented with additional analysis. Reads were aligned to a reference sequence (GRCh37), and sequence changes were identified and interpreted in the context of a single clinically relevant transcript. Enrichment and analysis focused on the coding sequence of the indicated transcripts, 20 bp of flanking intronic sequence and other specific genomic regions demonstrated to be causative of disease. Exonic deletions and duplications were called using an in-house algorithm that determines the copy number at each target by comparing the read depth for each target in the proband sequence with both mean read-depth and read-depth distribution. Confirmation of the presence and location of reportable variants was performed based on stringent criteria using one of several validated orthogonal approaches. A more detailed protocol of genetic testing and discrimination criteria is described in a previous paper [33].

### 5.2. Immunohistochemistry

To establish the presence and levels of expressions of E-cadherin, N-cadherin, SNAIL&SLUG, TWIST1, beta-catenin, GSK3beta and DVL1, immunohistochemistry was used. The samples were 4-μm FFPE sections fixed onto capillary gap microscope slides (DakoCytomation, Glostrup, Denmark). Immunohistochemistry protocol was described previously [29]. The antibodies and dilutions used are shown in Table 3.

Sections were immunostained using peroxidase/DAB+ (3,3-diaminobenzidine) (Dako REAL™ EnVision™, Glostrup, Denmark).

The level of expression in the healthy brain was determined by using the cerebral cortex of a human brain (Amsbio, Oxfordshire, UK) and data from Human Protein Atlas (https://www.proteinatlas.org). The frontal cortex of a healthy human brain, liver cancer, colon cancer tissue and normal bronchial epithelia all served as positive controls. Antibody labeling was analyzed by three independent observers blinded to the conditions of experiment using an Olympus BX53 microscope. ImageJ software (National Institutes of Health, Bethesda, MD, USA) was used to determine the cell number and the intensity of protein expression. In the field of view (magnification of 200×), we counted a minimum of 300 cells in tumor hotspot area and performed a semi-quantitative analysis, introducing the immunoreactivity score (IRS) to determine the signal strength. IRS is a factor that best correlates with computational photo analysis and was calculated by multiplying the percentage of cells with a positive signal in the sample (PP score) with staining intensity (SI score). PP score was determined as follows: no immunopositivity in tumor cells = score 0; 1–25% positive cells = score 1; >25–50% = score 2; >50–90% = score 3; >90% = score 4. The SI score was assessed in three categories mirroring the staining intensities: no staining or weak = score 1, moderate staining = score 2 and strong staining = score 3. The IRS score in our study ranged from 0–12. For the statistical analysis, the IRS values were converted to numbers/symbols: 1 (0/+) (IRS = 0–4), no expression or very weak expression; 2 (++) (IRS = 6, 8), moderate expression; 3 (+++) (IRS = 9, 12), strong expression.

## Figures and Tables

**Table 1:** PHD analysis of the first meningioma.

	Meningoma #1
Type	Grade I meningothelial meningioma
Location	Left frontotemporoparietal region
Consistency and dimensions	Soft consistency with dimensions 7.5 × 6 × 3 cm
Mitosis	Nuclei of tumor cells were hypochromatic. Only 1 mitosis found in 10 consecutive high-power fields.
Histological description	Nest configurations of meningothelial cells were placed in pseudosyncytial formation. Minor centers of necrosis were observed. Connective tissue, surrounding the tumor, was partially enlarged. A few psammoma bodies were spotted. On the margins of the specimen, there was a sharp transition from tumor cells to healthy brain tissue.

**Table 2:** PHD analysis of second meningioma.

	Meningoma #2
Type	Grade II atypical meningioma
Location	Right frontotemporoparietal region; tumor attached to dura
Consistency and dimensions	Grey, solid consistency with dimensions 7 × 4 cm
Mitosis	Maximum of 5 mitosis in 10 consecutive high-power fields were found
Histological description	The tumor was built of meningothelial cells forming a pseudosyncytial structure. Hypercellular areas with clear margins between cytoplasmic membranes were spotted. There was no necrosis inside the tissue. Hyalinization and calcification of connective tissue were seen in some areas. A sharp transition from tumor cells to healthy brain parenchyma tissue separated by layers of connective tissue.

**Table 3:** Antibodies and dilutions used for immunohistochemistry.

Antigen	Antibody	Type	Dilution
E-cadherin	E-cadherin clone: NCH-38 Code M3612 (Dako Santa Clara, CA, USA)	Monoclonal	1:100
N-cadherin	N-cadherin (D-4): sc-8424 (Santa Cruz Biotechnology, Inc. Dallas, TX, USA)	Monoclonal	1:200
TWIST1	Anti-Twist antibody [10E4E6] ab175430 (Abcam Cambridge, MA, USA)	Monoclonal	1:400
SNAIL&SLUG	Anti-SNAIL + SLUG antibody ab180714 (Abcam Cambridge, MA, USA)	Polyclonal	1:200
Beta-catenin (active)	Non-phospho (Active) β-Catenin (Ser33/37/Thr41), (D131A1), (Cell Signalling Technology, Danvers, MA, USA)	Monoclonal	1:800
Beta-catenin (total)	Clone b-Catenin-1, M3539 (Dako, Santa Clara, CA, USA)	Monoclonal	1:200
GSK3beta (active)	Anti-GSK3β (phospho Y216) ab75745 (Abcam, Cambridge, MA, USA)	Polyclonal	1:100
GSK3beta (inactive)	Anti-GSK3β (phospho S9) ab131097 (Abcam, Cambridge, MA, USA)	Polyclonal	1:100
DVL1	Anti-Dishevelled/Dvl1 antibody: ab233003 (Abcam Cambridge, MA, USA)	Polyclonal	1:200

## Data Availability

Data supporting reported results are contained within the article. Some of the data presented in this study are available on request from the corresponding author. The data are not publicly available due to privacy issues.
